# On the Local Employment of Chloroform in the Reduction of Dislocations

**Published:** 1860-05

**Authors:** 


*On the Local Employment of Chloroform in the Reduction of Dislocations.*—M. Orliac, a French provincial practitioner, relates two cases of recent dislocation of the shoulder, in which rapid and painless reduction was accomplished. This result he attributes to having surrounded the shoulder with, and placed in the axilla, compresses imbibed with ten or twelve grammes of chloroform, these being applied two or three minutes prior to, and during the attempt at reduction. In this way, he observes, assistants may be dispensed with (an important matter in country practice,) and pain be prevented, without any danger being incurred.—*Moniteur des Sciences Medicales.*

